# Transcriptome profiling of the honeybee parasite *Varroa destructor* provides new biological insights into the mite adult life cycle

**DOI:** 10.1186/s12864-018-4668-z

**Published:** 2018-05-04

**Authors:** Fanny Mondet, Andrea Rau, Christophe Klopp, Marine Rohmer, Dany Severac, Yves Le Conte, Cedric Alaux

**Affiliations:** 1 INRA, UR 406 Abeilles et Environnement, 84914 Avignon, France; 2 INRA, UMR 1313 GABI Génétique Animale et Biologie Intégrative, 78350 Jouy-en-Josas, France; 3 INRA, Genotoul Bioinfo, UR 875 MIAT Mathématiques et Informatique Appliquées de Toulouse, 31326 Castanet-Tolosan, France; 4 Institut de Génomique Fonctionnelle, UMR 5203 CNRS, U661 INSERM, Universités Montpellier 1 & 2, 34094 Montpellier, France

**Keywords:** *Apis mellifera*, Pollinator, Mite, Host-parasite interaction, Reproduction, Transcriptome, RNA-seq, Life cycle stages

## Abstract

**Background:**

The parasite *Varroa destructor* represents a significant threat to honeybee colonies. Indeed, development of *Varroa* infestation within colonies, if left untreated, often leads to the death of the colony. Although its impact on bees has been extensively studied, less is known about its biology and the functional processes governing its adult life cycle and adaptation to its host. We therefore developed a full life cycle transcriptomic catalogue in adult *Varroa* females and included pairwise comparisons with males, artificially-reared and non-reproducing females (10 life cycle stages and conditions in total).

**Results:**

Extensive remodeling of the *Varroa* transcriptome was observed, with an upregulation of energetic and chitin metabolic processes during the initial and final phases of the life cycle (e.g. phoretic and post-oviposition stages), whereas during reproductive stages in brood cells genes showing functions related to transcriptional regulation were overexpressed. Several neurotransmitter and neuropeptide receptors involved in behavioural regulation, as well as active compounds of salivary glands, were also expressed at a higher level outside the reproductive stages. No difference was detected between artificially-reared phoretic females and their counterparts in colonies, or between females who failed to reproduce and females who successfully reproduced, indicating that phoretic individuals can be reared outside host colonies without impacting their physiology and that mechanisms underlying reproductive failure occur before oogenesis.

**Conclusions:**

We discuss how these new findings reveal the remarkable adaptation of *Varroa* to its host biology and notably to the switch from living on adults to reproducing in sealed brood cells. By spanning the entire adult life cycle, our work captures the dynamic changes in the parasite gene expression and serves as a unique resource for deciphering *Varroa* biology and identifying new targets for mite control.

**Electronic supplementary material:**

The online version of this article (10.1186/s12864-018-4668-z) contains supplementary material, which is available to authorized users.

## Background

The parasitic mite *Varroa destructor* is currently considered to be the most significant pest of the western honeybee *Apis mellifera*, and no other pathogen has yet triggered a greater threat to apiculture [1, 2]. The *Varroa* mite is an ectoparasite of both adult and immature bees (brood), which can lead to the rapid death of infected colonies if left untreated [3].

*Varroa* relies entirely on its host for its own survival and propagation, with female adult life stages showing two distinct phases: the phoretic phase spent on adult bees, and the reproductive phase during which the mites reproduce in the brood [1, 4]. As adult bees emerge, female mites (mother and daughters) leave the brood cell and ride on adult workers or drones. During the time spent on bees, mites feed on hemolymph and often hide between the abdominal sternites of bees. Then, they start the reproductive phase by invading a brood cell containing a larva, shortly before the cell is capped. Once inside, the mite feeds on host hemolymph and lays a few eggs (first a male and then females). When sexually mature, the male mates with his sisters, which leave the brood cell with their mother upon adult bee emergence and start a new phoretic phase.

Pathological effects of *Varroa* are associated with the feeding activity of the mite on adult and developing bees. The mite is also associated, directly or indirectly, with several honeybee viruses, in particular with members of the deformed wing virus (DWV) complex [5–7], which might suppress bee immunity [8], although recent studies showed little evidence for immunosuppression [9, 10]. Since the transfer of *Varroa* from the Asian honeybee, *Apis cerana*, to the western honeybee, several control strategies have been developed to fight the mite and prevent colony losses. However, even the most successful approaches, e.g. acaricide treatments, currently raise serious concerns within the beekeeping community. Indeed, to control the *Varroa* population, beekeepers rely heavily on acaricides, such as pyrethroids (e.g.fluvalinate), amitraz, and coumaphos, which can lead to the emergence of acaricide-resistant strains as found for several compounds [11–13]. This highlights the urgent need for the development of new solutions to fight this detrimental parasite.

Progress in this direction has been rather slow, most likely due to limited knowledge on *Varroa* biology. In particular, little is known about the different physiological features characterising its adult life stages. However, recent technological advances have opened promising perspectives to help address these issues, in particular development in ‘omics technologies. An initial genome survey of *Varroa* was published in 2010 [14], and some studies have analyzed gene expression or protein profile changes in *Varroa*. For instance, transcriptomic analysis were performed on *Varroa destructor* synganglion [15] and foreleg (olfactory organ) [16]. A proteomic analysis was also performed on *V. destructor*, with a focus on the different developmental stages of the mite [17]. In addition, differential gene expression was investigated in *V. jacobsoni* with respect to the host on which they reproduce [18]. These studies completed the great progress made previously by Cabrera et al. in identifying and characterizing some genes associated to the phonetic and reproductive phases with a candidate gene approach [19–22]. However, there is a current lack of work on global physiological changes occuring during the adult life cycle of *Varroa*.

In this study, we investigated gene expression profiles of *Varroa* mites by sequencing total RNAs of seven adult life stages of the parasite. We present the first life cycle transcriptomic catalogue in adult *Varroa*, which provides a unique opportunity to identify key molecular features that characterise adult females, the most detrimental individuals of a *Varroa* mite population within a honeybee colony.

## Results and discussion

**Fig. 1:**
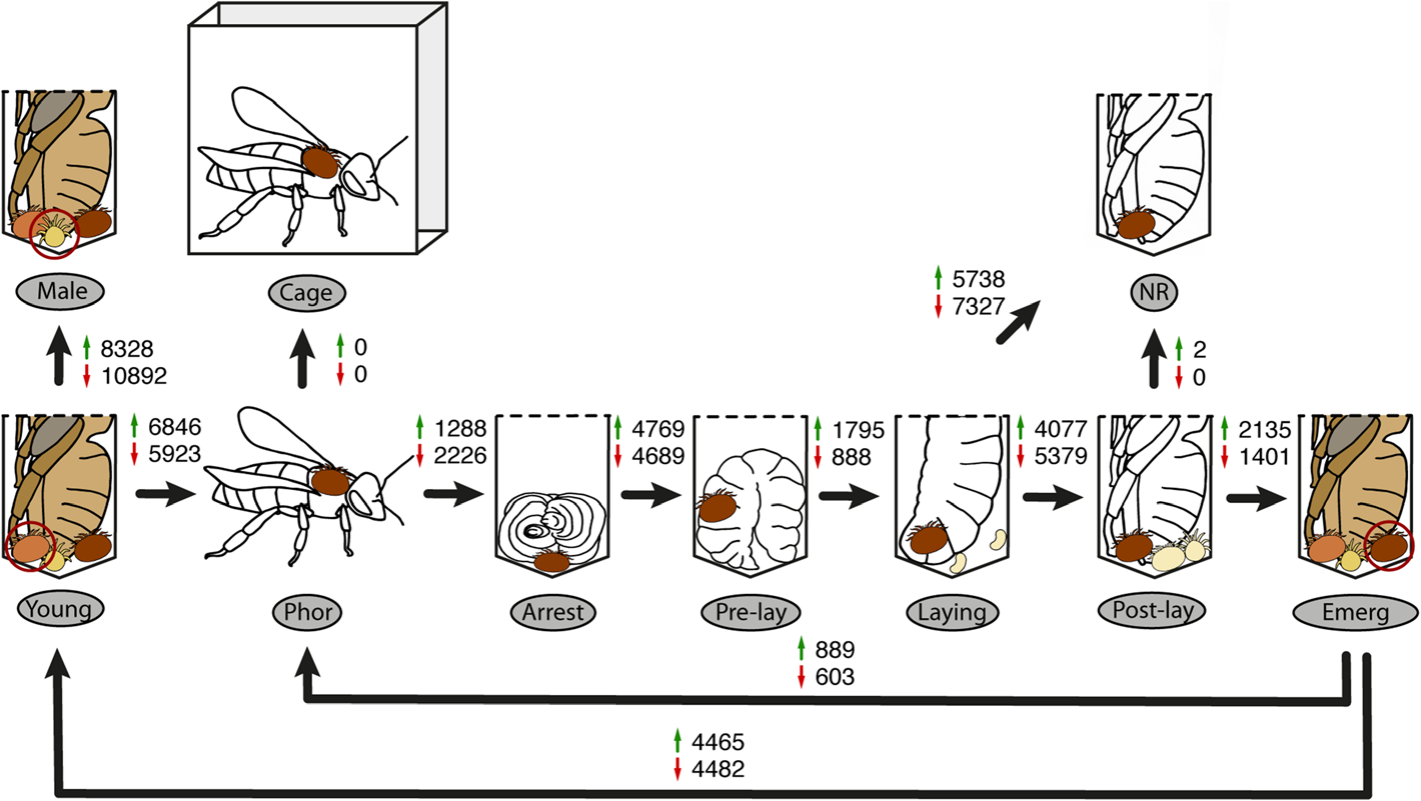
Differentially expressed contigs across the adult life cycle of *Varroa destructor* The adult life cycle of female *V. destructor* was analysed over 7 stages (bottom part) of the phoretic and reproductive parts of the life cycle. Three additional conditions were included for comparison (upper part): adult male (Male), artificially reared phoretic mite (Cage) and non-reproductive mite (NR). For each comparison between two stages indicated with black arrows, the number of up-regulated contigs is indicated with a green arrow while the number of down-regulated contigs is indicated with a red arrow (Benjamini-Hochberg adjusted *P*-values < 0.05). Young, young adult *Varroa* female in brood cells close to emergence; Phor, phoretic female on adult bees; Arrest, female mite in the arrestment stage at the bottom of brood cells ready to be capped; Pre-lay, female mite in freshly capped brood cells with visible signs of ovary activation; Laying, female mite laying eggs in pre-pupa brood cells; Post-lay, female mite rearing developing offspring in pupa brood cells (white bodied with dark eyed bees); Emerg, female mite at the end of the development period of its offspring in pupa brood cells (dark bodied with dark faced bees). Illustrations by Fanny Mondet.

To study the underlying molecular mechanisms governing the *Varroa destructor* adult life cycle, we carried out transcriptomic profiling of seven stages (Fig. 1) described in the Methods section and comprising: young mites (Young, collected from P8 to P9 cells), phoretic mites (Phor, collected on adult bees), arresting mites (Arrest, collected in unsealed L5 brood cells), pre-laying mites (Pre-lay, collected from sealed brood cells containing moving larva), laying mites (Laying, collected from sealed brood cells containing pre-pupae), post-laying mites (Post-lay, collected from capped brood cells containing purple-eye and white-body pupae P5), and emerging mites (Emerg, collected from P8 to P9 cells). In addition, we sampled non-reproducing mites (NR, collected from P5 brood cells, but without offspring), males (Male, collected from P8 to P9 cells), and phoretic mites artificially reared in cages with adult bees (Cage). A total of 40 RNA-seq libraries (*n* = 4 pools of 10 mites per stage or condition) were constructed and sequenced.

### De novo transcriptome assembly and functional annotation

A total of 1,154,425,396 raw reads, with an average of 28,860,635 ± 6,672,405 reads for each of the 10 life-cycle stages and conditions were generated. After removing 8531 low-quality reads and reads solely composed of sequencing adapters from the raw data set, a total of 1,154,416,865 reads were used for the transcriptome assemblies. The clean reads were assembled into 41,801 contigs ranging from 201 to 8610 bp (Additional file 1), with a median of 986 bp. The assembled transcriptome size is 30,107,462 bp.

Contigs were aligned with NCBI blast (blastx program, e-value under 1e-5) on Refseq, Swissprot and Ensembl protein databases from *Apis mellifera* and *Metaseiulus occidentalis*, which also belongs to the mite infraorder and has a fully assembled and annotated genome [23]. The assembly quality was then assessed in four ways: by calculating the read on contig re-alignment rate for every sample, by realigning the 308 *Varroa destructor* proteins found in NCBI protein database on the contigs, by determining the coverage and similarity of contigs on the 12,035 NCBI *Metaseiulus occidentalis* proteins, and last by processing the contigs with BUSCO (version 2) using the arthopoda_odb9 database [24]. This last method enables to check the presence and completeness of a set of expected single copy protein coding genes for a given branch of the evolution tree. The per sample read mapping rate mean was 85.73 ± 2.89% across libraries (Additional file 2). A total of 16,672 contigs could be annotated, from which 37.89% were annotated with reference to the sequences recorded in the RefSeq protein database (Additional files 3 and 4). Out of the 308 *Varroa destructor* proteins found in the NCBI protein database, 297 were present in the assembly (96.4%). The alignment coverage and identity reached 94.40 ± 19.53% and 88.57 ± 4.52%, respectively. The predatory mite *Metaseiulus occidentalis* was the species found most often in the RefSeq protein annotations, corresponding to 35.52% of the contigs (14,848 contigs). 8362 out of the 12,035 *M. occidentalis* proteins aligned on the contig set (69.42%). The alignment coverage and identity had mean values of 51.81 ± 31.41% and 77.66 ± 9.94%, respectively. The other species found as best hit annotations were the deer tick *Ixodes scapularis* (321 contigs) and the honeybee *A. mellifera* (269 contigs). Among the 1066 arthopoda_odb9 proteins searched by Busco V2, 851 were found complete in single or multiple copies. This represents 79.8% of the proteins expected in the genome. The remaining ones are missing (8.4%) or fragmented (11.8%).

### Transcriptomic profiles mirror stages of the adult mite cycle

First, we performed some initial exploratory analysis to check the overall reproducibility and variation between biological replicates belonging to the same life stage or condition. We generated a heatmap of Pearson correlations based on log_10_ expression counts to evaluate the sample relatedness and identify outliers (Additional files 5). Independent samples belonging to the same life stage were highly correlated. However, one sample from the Post-lay stage was identified as an outlier (R78–10) and was therefore removed from the downstream analysis. Male samples exhibited a higher similarity to each other than to female samples (Additional files 5).

**Fig. 2:**
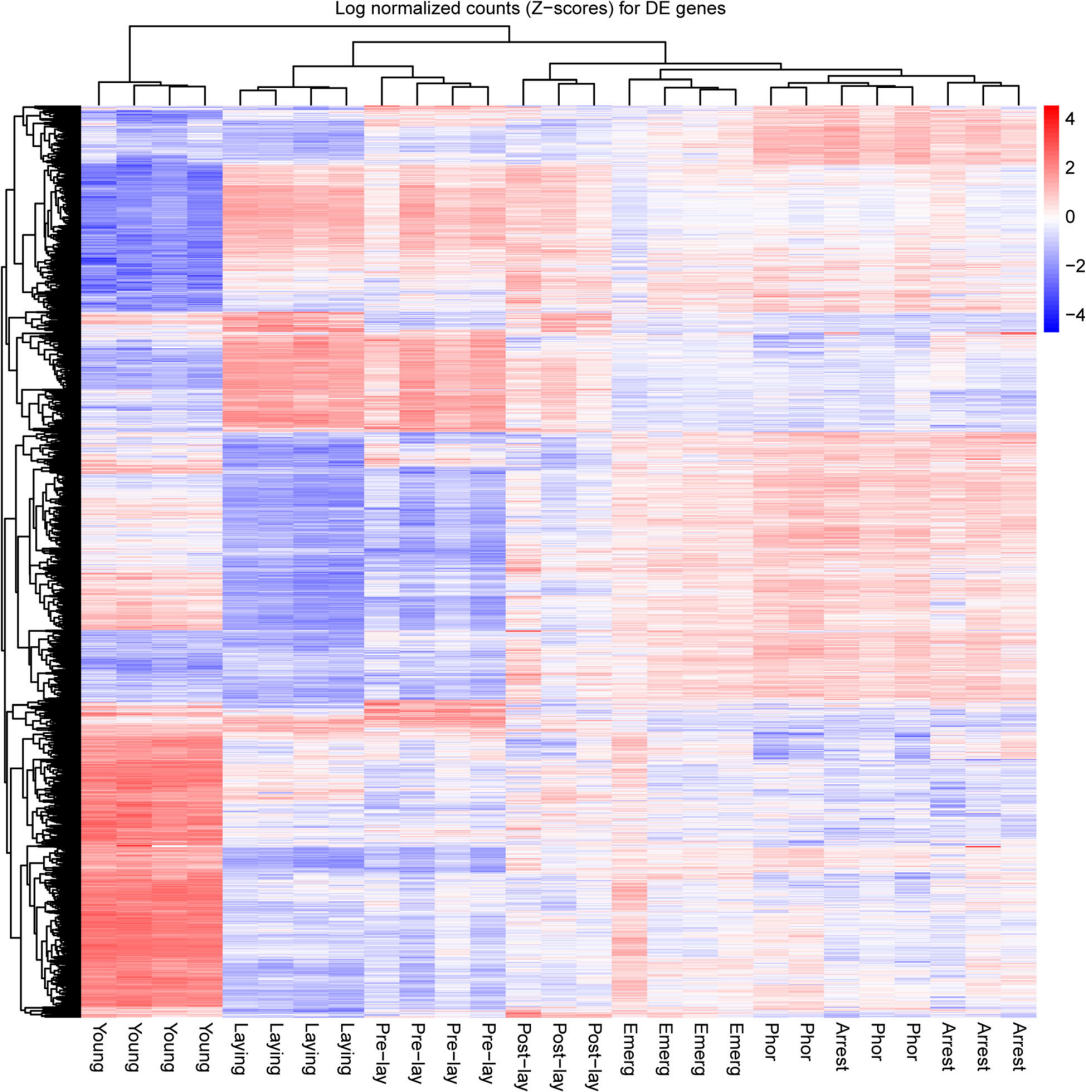
Heatmap and clustering of contigs identified as differentially expressed in one of the stage of the mite life cycle. Heatmap of the log normalized count *Z*-scores (i.e. for each contig the log-normalized count is centered to have mean of 0 and scaled to have variance of 1) for the 4005 contigs identified as differentially expressed in at least one of the following contrasts, using Bonferroni globally corrected *P*-values less than 5%: Young vs Phor, Phor vs Arrest, Arrest vs Pre-lay, Pre-lay vs Laying, Laying vs Post-lay, Post-lay vs Emerg

In order to visualise transcript expression patterns at the different stages during the adult cycle, we performed hierarchical clustering (Euclidean distance, complete linkage) on the log normalized count *Z*-scores for the 4005 differentially expressed contigs in at least one of the following contrasts (Bonferroni globally corrected *P*-values less than 5%): Young vs Phor, Phor vs Arrest, Arrest vs Pre-lay, Pre-lay vs Laying, Laying vs Post-lay, Post-lay vs Emerg. The hierarchical clustering analysis clearly segregated reproductive (Pre-lay and Laying) and non-reproductive stages (Post-lay, Emerg, Phor and Arrest) into distinct clusters (Fig. 2). A partitioning of non-reproductive stages into pre-reproductive *Varroa* (Phor and Arrest) and post-reproductive *Varroa* (Post-lay and Emerg) was also observed, which underlines distinct physiology and major changes induced by reproductive processes. Finally, newly-produced females (Young) were clearly segregated into a specific cluster. This clear distinction is likely due to an immature physiology. To further understand and extract biological meanings from these stage-specific transcript expressions, in the following sections we analyse and discuss pairwise comparisons and co-expression patterns across the adult life cycle.

### Transcriptome remodelling during *Varroa* life cycle

To capture the dynamic changes in gene expression during the *Varroa* life cycle, we focused on the following pairwise comparisons: Young vs Phor, Phor vs Arrest, Arrest vs Pre-lay, Pre-lay vs Laying, Laying vs Post-lay, Post-lay vs Emerg and Emerg vs Phor.

The *Varroa* transcriptome was found to constantly change during the cycle, however major changes were observed just before (Arrest vs Pre-lay) and after female reproduction (Laying vs Post-laying), indicating a profound physiological remodelling during the reproductive stages (Fig. 1 and Additional file 6). The smallest change in mite transcriptome was found between foundresses ready to emerge from the host cell after reproduction (Emerg) and phoretic females (Phor), suggesting that emerging mites were ready for a new phoretic phase. Finally, another major change was detected between young and phoretic mites, likely due to the physiological immaturity of young females.

### Vitellogenin, large lipid transfer protein and Halloween genes

To further assess the quality of our sampling, we specifically identified and analyzed the expression level of two genes involved in *Varroa* reproduction: vitellogenin 1 and 2 (*vg1* and *vg*2), both of whose expression peak during oviposition [21]. The expression of the large lipid transfer protein (*lltp*), exhibiting an opposite expression profile [19] was also checked. We observed a similar expression pattern, providing a validation of our sampling: a peak of expression of *vg1* and *vg2* during the Pre-lay and Laying stages and inversely a drop of *lltp* during those two stages as compared to other stages (Additional files 6 and 7).

Although it is well established in insects that the initiation of vitellogenesis and reproduction are regulated by juvenile hormone, biochemical evidence suggests that mites and ticks cannot synthetize this hormone [25, 26]. Instead, it is assumed that vitellogenesis is initiated by ecdysteroids in Acari [25, 26]). In insects, the final sequential hydroxylation of steroid precursors into active ecdysteroids are mediated by cytochrome P450s (CYP450s), encoded by Halloween genes [27]. Orthologs of these P450s have been identified in ticks and mites [23, 28] including *Varroa* [20]: spook (*CYP307a1*), disembodied (*CYP302A1*) and shade (*CYP314A1*). In a previous study, disembodied and shade were found to be more highly expressed during the reproductive phase as compared to the phoretic phase, but such an increase was not observed 4 h after the brood cell invasion [20]. Similarly, in our study no increase in the expression levels of spook, disembodied and shade was detected between the Phor and Arrest stages (Additional file 6). The expression level of shade did not change during reproduction but increased between the Young and Phoretic stages. However, we found that spook, disembodied and the ortholog of the mite *Metaseiulus occidentalis* ecdysone receptor were significantly upregulated in the Pre-lay vs Arrest stages and in the Laying vs Post-lay stages (except here for the ortholog of ecdysone receptor) (Additional files 6 and 8). Altogether, those results suggest that Halloween genes in *Varroa* are transcriptionally upregulated to initiate the synthesis of ecdysteroid and reproduction.

### Biological features of *Varroa* life cycle

**Fig. 3:**
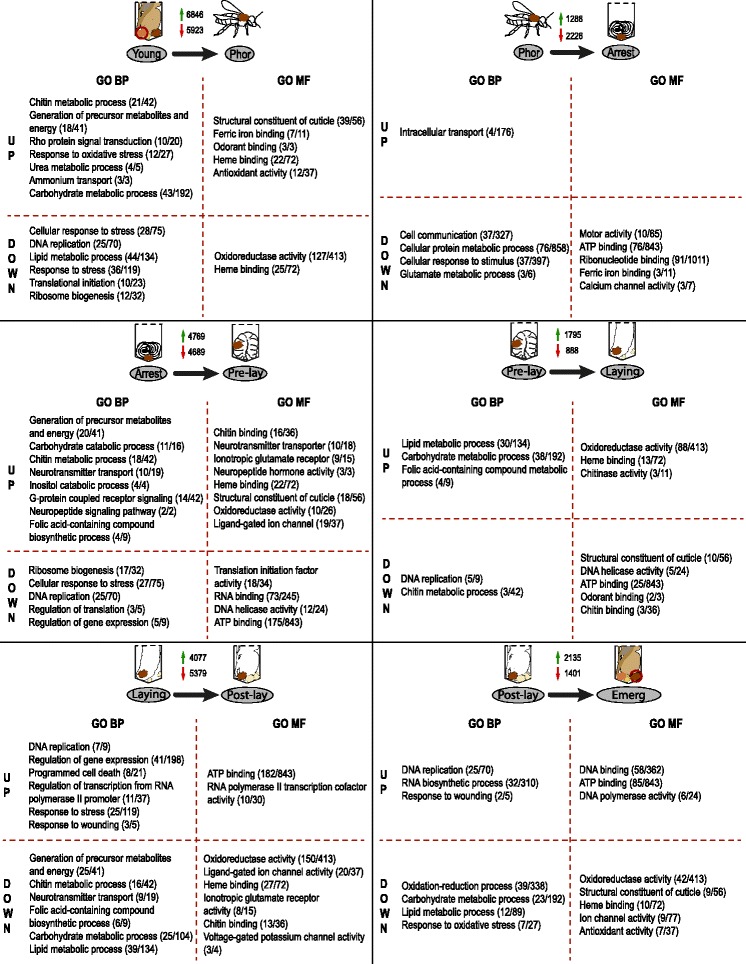
GO analysis of pairwise comparisons of conditions across the adult life cycle of *Varroa* females. Six comparisons of conditions across the female adult life cycle are shown. For each comparison, selected GO functions of interest are presented. Numbers in brackets indicate the number of significant contigs corresponding to a given function, followed by the number of annotated contigs in this comparison. BP, Biological Process; MF, Molecular Function; UP, upregulated; DOWN, downregulated. Illustrations by Fanny Mondet

Gene Ontology (GO) enrichment analysis was performed to explore which functional components are involved in the *Varroa* life cycle. The corresponding lists of GO functions (biological process and molecular functions) for each pairwise comparison are shown in Additional file 9. Several functions changed between each stage but globally we found that functions like generation of precursor metabolites and energy, carbohydrate metabolic process, lipid metabolic process, heme binding and chitin binding or chitin metabolic process followed the same pattern of regulation, being upregulated in young *Varroa* females and then decreasing until oviposition (Laying stage), from which it increased again until foundress mites were ready to emerge from the brood cell and become phoretic again (Fig. 3). Functions like DNA replication, regulation of translation and ribosome biogenesis exhibited an opposite expression pattern, being most representative of reproductive stages (Pre-lay and Laying). Since the ability to reproduce requires high levels of energy, an overrepresentation of functions linked to energetic processes was expected during foundress reproduction in brood cells. For example, energetic metabolism was found to be one of the most significantly enriched physiological processes in foundresses (adult) when compared to other developmental stages (protonymph, deutonymph) [17]. However, such functions were rather upregulated before and after reproduction (egg-laying). This is in accordance with the study of Garedew et al. [29] on the energy and nutritional demands of foundresses. The authors showed that foundresses in brood cells have a low metabolic rate and are very inefficient in energy use. They thus have to feed constantly to fulfil their energetic requirements. Indeed, vitellogenesis is initiated via the acquisition by oocytes of nutrients from the ovarian and lyrate organ tissues [30, 31] and from the host hemolymph [32, 33] once the female has started to feed on a 5th instar larva (Pre-lay). For example, some of the hemolymph proteins are ingested from the host and directly sequestered in eggs without any proteolytic digestion [32, 34]. Altogether, those observations suggest that nutrients are directly acquired by eggs from the host and female’s own storage, likely with little metabolic activity. The energetic metabolism peak observed before and after reproduction might in that case correspond to nutrient acquisition, metabolisation and storage in ovaries and dedicated organs to sustain future egg production.

Chitin binding and chitin metabolic process were functions enriched in young females characterized by a soft and light body coloration as compared to mature females (Fig. 3). Chitin is the main structural component of arthropod cuticle, which provides mechanical support and an effective barrier to desiccation and infections. The overexpression of chitin processes in young females would suggest cuticle hardening, as evidenced by the significant upregulation of the gene encoding the neuropeptide bursicon when compared to phoretic females. In insects, this neuropeptide is released after the ecdysis completion and triggers the tanning (melanisation and sclerotization) of the new cuticle [35]. This is in accordance with a proteomic study of *Varroa* developmental stages, which reported an upregulation of chitin metabolism in nymphs as compared to adults likely for the exoskeleton formation [17]. Interestingly, the functions related to chitin metabolism were downregulated during oviposition in brood cells (Pre-lay and Laying stages; Fig. 3). In the ixodid ticks, the synthesis of flexible cuticle enables its distension during ingestion of a large blood meal (around 100-fold increase in size of the individuals) [28]. A link between feeding behaviour and cuticle synthesis was thus expected. However, *Varroa* foundresses can ingest large amounts of hemolymph during reproduction and utilize up to 25% of the nutritional reserves of pupae [29]. An alternative hypothesis would be that, when reproducing, females invest less energy in cuticle synthesis since in capped brood cells they are less exposed to temperature and humidity variation and protected from grooming behaviours by adult bees, which can damage their cuticle [36]. *Varroa* females would therefore perfectly adapt their investment in chitin metabolism according to their host environment.

Heme binding was also markedly overrepresented in individuals outside the reproductive window (Fig. 3). Enzymes that bind iron and heme are involved in several key biological functions, including oxygen transport and sensing, synthesis of DNA, ecdysone and lipids [37]. Although it seems that hematophagous ticks are incapable of de novo heme biosynthesis [38], several steps in the heme synthesis pathway have been found in *Ixodes scapularis* [28]. Likewise, we also found some steps of this pathway in the *Varroa* transcriptome: aminolevulinate synthase, aminolevulinic acid dehydratase, porphobilinogen deaminase, uroporphyrinogen-III synthase, uroporphyrinogen decarboxylase, protoporphyrinogen oxidase, ferrochelatase, protoheme IX farnesyltransferase. However, in *I. scapularis*, Gulia-Nuss et al. suggested that those genes might be remnants of a once functional heme synthesis pathway until adaptation to blood feeding [28]. In hematophagous insects and ticks, iron is required for optimal egg development and viable offspring, and heme sequestration by heme-binding proteins co-evolved with blood feeding [28, 39]. We could therefore speculate that the increase in heme-binding protein before and after reproduction might reflect an increase in iron sequestration and prepare the organism for egg production, as observed for the energetic metabolism.

Finally, functions related to DNA replication, transcription and translation were key features of foundresses during reproduction in brood cells (Pre-lay and Lay stages), and were likely connected to oogenesis and cell division (Fig. 3). Indeed, the oocytes maturation in the ovary is characterized by an increase in nucleic acid, as observed with toluidine blue staining in *Varroa* females [40]. Expression levels of transcription factor were also found to be related to reproduction in *V. jacobsini* [18]. Interestingly, the GO function folic-acid containing compound metabolic process was enriched during the Arrest and Pre-lay stages (upregulated in Arrest vs Pre-lay stages and Pre-lay vs Laying stages). Folic acid is an important vitamin for animals and must be present in the diet for normal growth and development, as it normally cannot be synthetized. When converted to folate forms, it is used in DNA synthesis and amino acid metabolism, which are required for cell division. As an illustration of its biological importance, it has been reported in several insect species that individuals are more viable and fecund when fed with a diet containing folic acid than individual lacking this vitamin [41]. *Varroa* likely consumes folic acid from its host or potentially harbors some bacterial symbionts that provide essential folates [41]. The observed sharp increase of folic acid metabolism during female reproduction indicates that it is also essential for *Varroa* fecundity by sustaining DNA synthesis and cell division.

### Male vs female *Varroa*

**Fig. 4:**
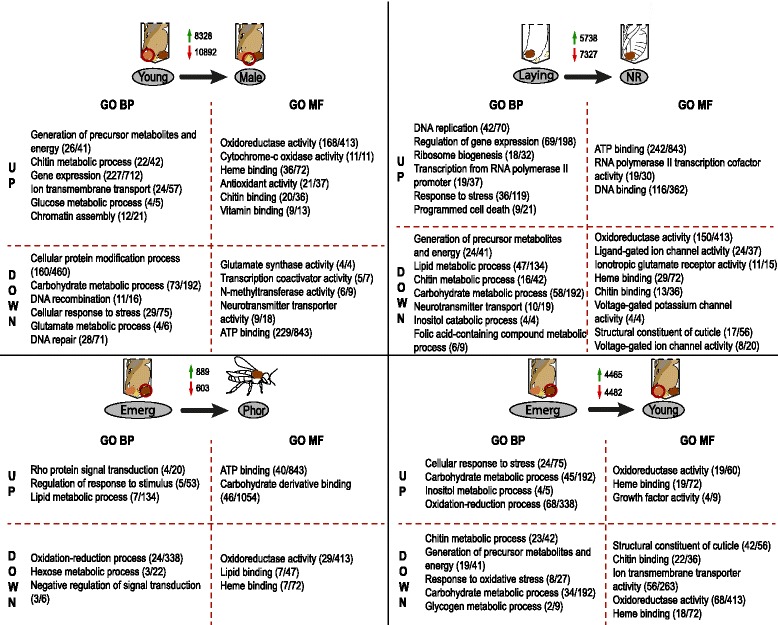
GO analysis of pairwise comparisons of selected conditions of the adult life cycle of *Varroa* mites. Four comparisons were included: young adult female versus male, laying female versus non reproductive female, female in cell ready to emerge versus phoretic mite, young versus females that have reproduced at least once. Two comparisons were not included as no or only 2 contigs were identified as differentially expressed: phoretic mite naturally reared versus reared in cages, post-laying female versus non-reproductive female, respectively. For each comparison, selected GO functions of interest are presented. Numbers in brackets indicate the number of significant contigs corresponding to a given function, followed by the number of annotated contigs in this comparison. BP, Biological Process; MF, Molecular Function; UP, upregulated; DOWN, downregulated. Illustrations by Fanny Mondet

When looking at pairwise comparisons, the largest difference in the number of differentially expressed contigs was found between haploid males and diploid females. In their proteomic study, McAfee et al. found a large fraction of proteins (over 80%) to be upregulated in males as compared to females [17]. We did not observe this pattern (43% upregulated in males, Additional file 6). Besides the level of analysis (proteomic vs transcriptomic), the major difference between those two studies come from the type of samples used for the comparative analysis: pools of deutonymphs, adult daughters and foundresses versus pools of deutonymphs and adult sons in McAfee et al. [17], and adult sons versus adult daughters in our study. Nevertheless, like in McAfee et al. [17], we found enrichment of functions related to chromatin assembly, energetic metabolism and gene expression among the genes differentially expressed between males and females (Fig. 4 and Additional file 9). More precisely, energetic metabolism and chromatin assembly were upregulated in females, and functions like DNA replication and repair were downregulated. In addition, chitin metabolism process was specifically enriched in females, demonstrating the active exoskeleton formation and hardening in females as compared to males. In the proteomic studies, histone lysine N-methyltransferase was in the top 10 of proteins that were the most differentially expressed between males and females. Similarly, in the honeybee ectoparasite *Tropilaelaps mercedesae*, the comparison between adult male and female transcriptomes showed that histone-lysine-*N*-methyltransferase gene family was more highly expressed in males than in females [42]. This is supported by our data revealing an overrepresentation of the function related to N-methyltransferase activity in males as compared to females. The methylation of histones for the epigenetic modification of chromatin and the regulation of gene expression might therefore be a conserved mechanism involved in sexual differentiation and maybe dosage compensation [43]. The ortholog E3 ubiquitin-protein ligase HERC4 was one of the most differentially expressed gene between males and females. Its disruption in mice is known for causing defects in spermatozoon maturation and impairing fertility [44]. Finally, many contigs of spermatogenesis-associated proteins were upregulated in males. Those proteins, as their name indicate, act in sperm development in mammals as well as in insects [45]. Suppression of their expression in the brown planthopper *Nilaparvata lugens* leads to decreased male accessory gland protein content and reproductive system development, as well as defects in the reproductive biology of their mating partners, providing good candidates for RNAi based control of insect pests [46].

### Reproductive vs non-reproductive *Varroa*

It has been known for a long time that a certain percentage of female mites fail to reproduce after invading a brood cell [1]. Therefore, unravelling the mechanisms that trigger reproductive failure represent a crucial step for paving the way towards a better control of mite population within colonies. For that purpose, we compared here the transcriptomes of females who successfully reproduced and others that failed**.** Only two contigs were differentially expressed between the two female types; with one encoding a metalloproteinase (Additional file 6). In addition, the set of functions that were overrepresented between the pairwise comparisons: egg-laying females vs post-laying females and egg-laying females vs non-reproducing females were highly similar and related to energetic metabolism, chitin metabolism (downregulated in laying females) and regulation of gene expression (upregulated in laying females) (Additional file 9 and Figs. 3 and 4). The GO function folic-acid containing compound metabolic process was enriched in both cases after egg-laying, likely preparing the organism for a new reproductive cycle. In sum, reproductive and non-reproductive females were highly similar and it was not possible to directly distinguish them. Since non-reproductive and reproductive females have a very similar profile, it seems that mechanisms that inhibited *Varroa* reproduction occurred before oogenesis. For instance, a recent study revealed that successful *Varroa* reproduction is determined by a specific time frame: mite oogenesis is stimulated by larva kairomones and occurs within 12 h after worker brood cells are capped; after that another set of chemicals seems to interrupt mite oogenesis [47]. A bad timing and as a consequence failure in oogenesis initiation could therefore prevent *Varroa* reproduction. However, we cannot exclude the existence of detrimental factors from the host (e.g. immune defense) or the parasite itself (e.g. low levels of nutrients) that prevented mite oogenesis.

### Mother vs daughter *Varroa*

The major changes between mother (Emerg) and daughter (Young) transcriptomes concern functions related to carbohydrate metabolic process, structural constituent of cuticle, and the generation of precursor metabolites and energy, which were upregulated in daughters (Fig. 4 and Additional file 9). Those changes are consistent with the cuticle hardening and physiological maturation operating in young *Varroa* females. The function cellular response to stress was enriched in mother mites, possibly as a result of cellular changes in state and activity following oviposition, as also observed between egg-laying (Laying stage) and post-laying females (Post-laying stage) (Fig. 4). Interestingly, the contigs related to the cholecystokinin receptor type A were upregulated in mothers (Additional file 6). This neuropeptide regulates many behavioural functions in insects, like the inhibition of food intake and the stimulation of hyperactivity and locomotion [48]. If cholecystokinin functions are conserved in *Varroa* mites, it could reflect behavioural differences between mothers, ready to start a new phoretic stage once they reproduced, and daughters, who needs to feed and stay in the cells in order to complete their physiological and morphological development.

### Artificially-reared phoretic *Varroa*

Many efforts are currently being made to improve our knowledge of *Varroa* biology and life cycle, as well as to identify chemical treatments again this parasite. For this purpose, many studies have attempted to either rear the mite on its host or not under laboratory conditions [49–52]. In order to assess whether or not artificial rearing conditions are stressful and induce significant changes in *Varroa* physiology, we artificially-reared phoretic *Varroa* on its host for 4 days under laboratory conditions and compared their transcriptome to colony-collected phoretic *Varroa*. No significant change was observed between both rearing conditions (Additional file 6), which demonstrates the suitability of such rearing conditions for studying *Varroa* biology or resistance to chemical treatments, at least at the phoretic stage.

### Bee transcripts in *Varroa*

We found contigs encoding honeybee hexamerin 70b, 70c, 70a and 110, transferrin and apolipophorin in female mites and reaching a peak at the Arrest stage - mites in unsealed L5 brood cells (Additional files 6 and 10). The presence of honeybee hemolymph proteins, including hexamerin, transferrin and apolipophorin, has already been reported in *Varroa* [17, 53], and can provide an estimate of the undigested protein diet of *Varroa*. Since such proteins are synthetized in the bee fat body (before being released in hemolymph), it might be surprising to find their transcripts in mites. However, the levels of hexamerin, transferrin and apolipophorin, which are involved in the juvenile hormone-binding protein molecular traffic, reach a peak at the end of the L5 instar [54, 55]. Furthermore, *Varroa* might have a better access to the fat body of larva than adults, as evidenced by the fact that transcripts of bee vitellogenin, which is also synthesised in the fat body and is at a higher level in adults than in larvae (at least 50-fold difference) [56], were not found in *Varroa*. Altogether, those observations provide the most parsimonious explanation for the presence of bee transcripts in *Varroa* at the Arrest stage.

### Co-expression analysis of the *Varroa* life cycle

**Fig. 5:**
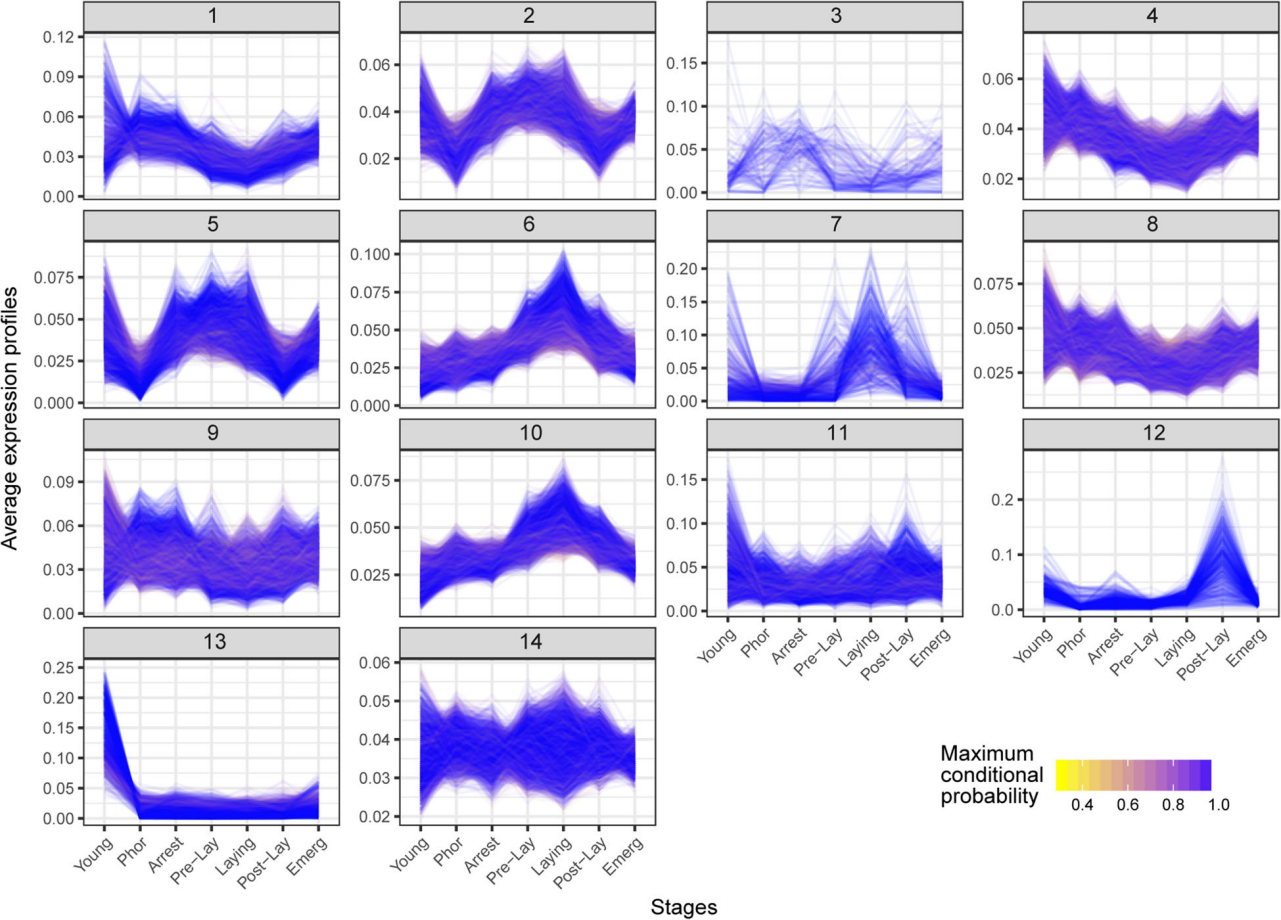
Co-expression analysis of *Varroa* mite life cycle. Normalized expression profiles and cluster assignment of contigs that were differentially expressed in at least one of the following contrasts, using BH corrected *P*-values less than 10%: Young vs Phor, Phor vs Arrest, Arrest vs Pre-lay, Pre-lay vs Laying, Laying vs Post-lay, Post-lay vs Emerg. Fourteen clusters were identified. The colour scale represents the maximum conditional probability of cluster membership for each contig, with yellow and blue representing weak and strong probabilities, respectively

**Fig. 6:**
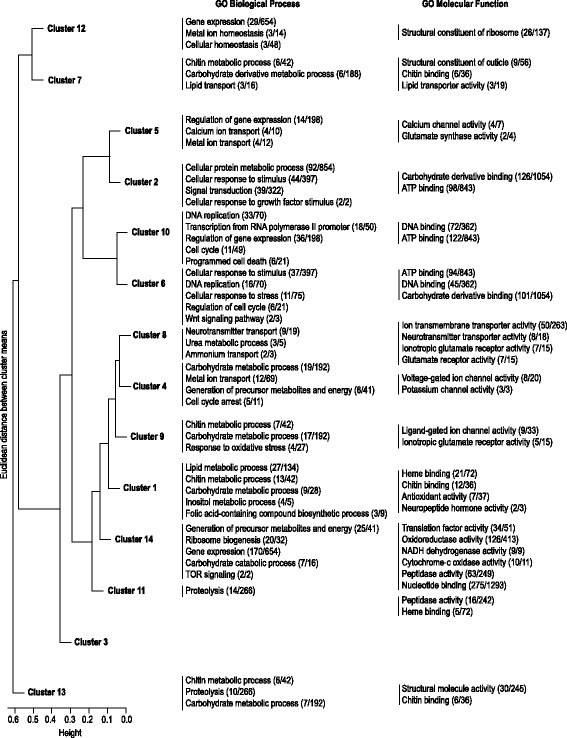
Hierarchical clustering and GO analysis of clusters revealed by the co-expression analysis. Fourteen clusters were identified and compared through a hierarchical classification. For each cluster, selected GO functions of interest are presented. Numbers in brackets indicate the number of significant contigs corresponding to a given function, followed by the number of annotated contigs in this comparison

The next objective was to identify genes and biological processes associated with the progression of *Varroa* life cycle using differential expression and co-expression analysis. We performed a co-expression analysis for contigs identified as differentially expressed in at least one of the comparisons involving the life-cycle stages (Young, Phor, Arrest, Pre-lay, Laying, Post-lay, Emerg; Benjamini-Hochberg (BH) adjusted *P*-value < 0.10) and identified 14 clusters, with most of them demonstrating a stage-specific expression (Fig. 5 and Additional file 11). For instance, the cluster 13 was primarily expressed in the Young stage, and the clusters 6 and 10 in the Laying stage. Some clusters were visually similar, which was confirmed by a hierarchical clustering analysis on the cluster profiles revealed by the co-expression analysis (Fig. 6). Clusters 2, 5, 6 and 10, which were mostly expressed during brood cell invasion and foundress reproduction, were segregated from clusters 1, 4, 8 and 9 showing a pre- and post-reproduction specific expression. The other clusters (3, 7, 11, 12 and 14) did not exhibit a clear stage-specific expression pattern. The GO enrichment analysis on contigs related to each cluster highlighted the same biological features revealed by the pairwise comparisons (Fig. 6 and Additional file 12**)**. On one hand, in clusters 2, 5, 6 and 10, which were specific to reproductive stages, the most enriched functions were related to the regulation of gene expression, cell cycle and cellular response to stimulus (Fig. 6). On the other hand, in clusters 1, 4, 8 and 9, which were specific to pre- and post- reproductive stages, processes linked to energetic metabolism, chitin binding, heme binding, and ionotropic glutamate receptor activity were among the most representative.

In order to get some more insight on the biological processes underlying *Varroa* life cycle we looked for orthologs of *Ixodes scapularis* (tick vector of Lyme disease) and *Metaseiulus occidentalis* (predatory mite) genes involved in functions and pathways that *i)* are important to the tick and mite biology [23, 28] and *ii)* show a specific expression pattern during the *Varroa* life cycle based on the co-expression analysis (i.e. identified in one of the co-expression clusters).

#### RNA interference machinery

**Fig. 7:**
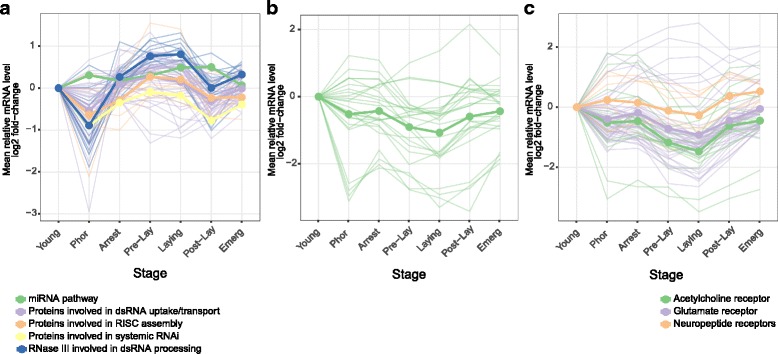
Dynamic expression of contigs encoding genes from the RNAi pathways (**a**), sialome (**b**) and neurotransmitters and neuropeptides receptors **(c).** Only contigs that were identified in clusters from the co-expression analysis are shown. Contig IDs are shown in the Additional file 13. After averaging values for each contig across replicates in each stage, mean expression values are reported as log_2_ fold-change with respect to the Young stage. Thick lines represent the average expression of contigs belonging to the same functional category

Orthologs of the RNA interference (RNAi) pathways were found to be differentially expressed across the life cycle. This conserved pathway is activated in response to both endogenous and exogenous pathogenic nucleic acids (double stranded RNA) and regulate the expression of protein-coding genes [57, 58]. Most of the contigs of this pathway followed the same pattern of expression and were upregulated during reproduction (Arrest, Pre-lay and Laying stages) (Fig. 7a and Additional file 13). RNAi is an important defence against viruses [59]; however, no obvious increase or change in virus levels was observed during the life cycle (see below and Additional file 14). In addition, immunity and reproduction, which are energetically demanding processes, have often been found to trade-off in female insects [60]. The increased expression of RNAi pathways does not seem to be associated with an immune response here, but might instead reflect a rise in transcriptional regulation activity as evidenced by the enrichment of functions related to the regulation of gene expression during reproduction (see above).

#### Sialome

When feeding, the *Varroa* female punctures its host cuticle with its chelicerae, injects saliva into the hemolymph through the open wound, and then ingests bees’ hemolymph. Interestingly, the expression level of genes from the sialome (set of mRNA and proteins expressed in salivary glands) abruptly decreased in *Varroa* females during reproduction (Fig. 7b and Additional file 13). In blood sucking animals, the saliva contains a complex mixture of compounds that disarm the host’s hemostasis, inflammation and immunity, therefore facilitating the blood feeding [61, 62]. In addition, salivary glands can produce digestive enzymes involved in digestion of skin matrix constituents [62]. Since females regularly feed on adult and immature hosts, the reason for such a decrease in the sialome expression during reproduction is not clear. To our knowledge, it is not known whether adult bees are more resistant to wounding (better hemostasis, inflammation and immunity) than immature bees, but it is possible that *Varroa* adapts to its host physiology and morphology during its cycle. For example, during reproduction *Varroa* feeds on larvae and pupae that have a flexible and soft unsclerotised cuticle, as compared to the rigid cuticle of adult bees. Therefore, *Varroa* might invest less energy in saliva secretion during reproduction assuming that immature bees represent an easier meal for them at the enzymatic level.

#### Neurotransmitters and neuropeptides receptors

We found a common expression profile for orthologs of glutamate, acetylcholine and several neuropeptides receptors, characterized by a marked drop in their expression levels during foundress reproduction (Pre-lay and Laying stages) (Fig. 7C and Additional file 13). Glutamate and acetylcholine are neurotransmitters playing a key role in neural transmission in animals. In insects, acetylcholine is the most abundant neurotransmitter in the brain and is particularly involved in sensory pathways and specifically in the olfactory system [63], and glutamate is notably implicated in both smell and taste [64]. Based on this knowledge, our results logically suggest that *Varroa* females are more sensitive to environmental stimuli before and after reproduction, likely to perform host-finding behaviours (discriminate between nurses over foragers and detect new brood cell at L5 ready to invade) [1, 4, 65]. The expression pattern of receptors to cholecystokinin, capa neuropeptides and neuropeptides Y (homolog of neuropeptide F in insects) might also be involved in the regulation of the adult life cycle and notably the transition from the phoretic to reproductive phase and the inverse. Indeed, in *Drosophila* the neuropeptide F and cholecystokinin are bioactive messenger molecules involved in a wide array of vital biological processes like foraging, feeding, stress, aggression, learning, and locomotion [48, 66]. In *Varroa*, their functional role is not known but they are good candidates for the regulation of the more complex behaviours exhibited by phoretic females than reproductive females in brood cells. Last but not least, the capa neuropeptide decreases desiccation tolerance but shortens chill coma recovery time in fruit fly [67]. Such physical parameters are important in *Varroa* biology since females are very sensitive to high relative humidity and almost never reproduce above 80% relative humidity [68]. Furthermore, *Varroa* are more likely to be exposed to temperature variation during their phorectic phase than during reproduction in sealed brood cells that are thermoregulated by adult bees.

### Viruses

A total of 30 contigs matched 4 of the 22 honeybee virus species previously screened [7]. They aligned to DWV (Deformed wing virus), VDV-1 (*Varroa destructor* virus 1), VDV-2 (*Varroa destructor* virus 2), and Macula-like virus, with 4, 12, 12 and 2 contigs representing each species, respectively. VDV-1 was the most abundant virus across all stages (Additional file 14).

Pairwise comparisons for each virus revealed no significant difference in abundance between the different stages/conditions, except for VDV-2 for which differences were identified between several stages (ANOVA, Tukey adjusted *P*-values < 0.05): Young vs {Cage, Male}, Phor vs {Pre-lay, Laying, Male}, Arrest vs {Pre-lay, Lay, Male}, Pre-lay vs {Post-lay, Cage, Male}, Laying vs {Post-lay, Cage, Male}, Post-lay vs Male, Emerg vs {Cage, Male}, NR vs Male, Cage vs Male. The small number of differences identified is most likely due to the high variability in virus abundance between biological replicates. This result however suggests that no stage of the adult life cycle of *Varroa* is particularly sensitive to virus infections and/or represents a higher threat to bees regarding virus loads.

## Conclusion

Our study provides the first full life-cycle transcriptomic catalogue in adult *Varroa* and reveals key genes and biological processes that are involved in the physiological, behavioural and functional changes occurring through the different stages. Specifically, our data indicate that functions related to energetic and chitin metabolic process were highly active during the initial phases of the cycle (e.g. Phoretic phases), whereas transcriptional regulation dominated in the subsequent phases (e.g. Pre-lay and Laying phases). Furthermore, several neurotransmitter and neuropeptide receptors involved in behavioural regulation, as well as sialome compounds, were greatly upregulated outside the reproduction stages. Altogether, those findings highlight the remarkable adaptation of the parasite to its host, especially to its different development stages and associated environments (free-moving adults vs pupae in sealed cells). This work also argues for the value of transcriptomic data to derive new insight into the parasite biology (e.g. biological inferences gained from gene ontology and functional enrichment analysis) and potentially identify new targets for *Varroa* control. Finally, this publicly available transcriptomic dataset is intended to provide a resource for the research community to extend the exploration of *Varroa* biology beyond the scope of this study.

## Methods

### Collection of *Varroa* mite sample

This study was performed using *Apis mellifera* L*.* honeybee colonies naturally infested by *Varroa destructor* mites, in the summer of 2015. Colonies were located at the INRA (Institut National de la Recherche Agronomique) research centre of Avignon (France). Adult mites were collected from 4 unrelated colonies, and were sampled according to their life stage. Seven life stages were considered, and identified thanks to characteristics of mites and/or hosting bees (Fig. 1). Except for phoretic mites, all stages were sampled from brood frames removed from the colonies and dissected in the laboratory. Two additional particular conditions and males were included for comparison of specific physiological features.

1. Phoretic mites: capped brood frames of each colony were removed to prevent any mite being released from emerging brood cells. Four days later, one frame of bees was removed from the colony and phoretic mites were sampled directly off adult bees.
2. Phoretic mites artificially reared: An additional 20 mites were sampled together with the bees they were standing on and placed in a plastic cage (10.5 × 7.5 × 11.5 cm), with 10 other non-infested bees. Cages were kept for 4 days in an incubator (28 °C) before mites were sampled.
3. Arresting mites: these mites were collected in unsealed L5 brood cells, as mites seen immobile at the bottom of the cells, immersed in larval food.
4. Pre-laying mites: these mites were collected from sealed brood cells containing moving larvae. These mites displayed an inflated ventral side due to activation of the ovaries.
5. Laying mites: these mites were collected from sealed brood cells containing pre-pupae and at least one mite egg (but no mite protonymph).
6. Post-laying mites: these mites were collected from capped brood cells containing purple-eye and white-body pupae (P5). Female mites were sampled if they were accompanied by at least one male and one female deutonymphs.
7. Non-reproducing mites: these mites were also collected from P5 brood cells, but identified as female mites with no offspring, i.e. in reproduction failure.
8. Emerging mites: these mites were collected from capped brood cells containing developing bees almost ready to emerge (but with an intact cell cap – P8 to P9).
9. Young mites: these mites were also collected from P8 to P9 cells, but identified thanks to their lighter body coloration as compared to founder females. These mites could have just mated, but have yet to undergo their first phoretic and reproductive stages.
10. Male mites: these mites were also collected from P8 to P9 cells and identified thanks to their striking sexual dimorphism as compared to females. Males exhibit a yellow tan and a round-body shape.

Mites were sampled using paint brushes, placed in 2 mL microcentrifuge tubes, and stored at − 80 °C. Transcriptomic analyses were performed on pools of 10 individual mites sampled from the same colony (*n* = 4 colonies, giving 4 pools per stage and condition).

### RNA isolation

Each sample of 10 mites was homogenised in 500 μL of Trizol® with a TissueLyser and 7 mm stainless beads (Qiagen, Courtaboeuf, France) (4 × 30 s at 30 Hz). After 5 min of incubation at room tempearture, 100 μL of chloroform was added. Samples were vortexed for 15 s and then left for 3 min at room temperature. They were then centrifuged at 12,000 rcf for 15 min at 4 °C. The upper phase was gently pipetted out. Total RNA was extracted following the QiagenUniversal Mini Kit instructions, and eluted in 30 μL nuclease-free water. RNA yield and concentration was determined using a Nanodrop (Thermo Fisher Scientific, Villebon-sur-Yvette, France) and samples were stored at − 80 °C until further processing.

### Transcriptome sequencing

RNA-seq libraries were constructed with the Truseq stranded mRNA sample preparation (Low throughput protocol) kit from Illumina (San Diego, CA, USA). Two hundred nanograms of total RNA were used for the construction of the libraries. The first step in the workflow involved purifying the poly-A containing mRNA molecules using poly-T oligo attached magnetic beads. Following purification, the mRNA fragments were copied into first strand cDNA using SuperScript IV reverse transcriptase (Thermo Fisher Scientific), Actinomycine D and random hexamer primers. This process reverse transcribes the cleaved RNA fragments that were primed with random hexamers. The addition of Actinomycin D to the first stand synthesis mix prevents spurious DNA-dependent synthesis, while allowing RNA-dependent synthesis, improving strand specificity. The second strand cDNA was synthesized using DNA Polymerase I, Rnase H and by replacing dTTP with dUTP. This process removes the RNA template and synthesizes a replacement strand. The incorporation of dUTP quenches the second strand during amplification because the polymerase does not incorporate past this nucleotide. These cDNA fragments then had the addition of a single ‘A’ base and subsequent ligation of the adapter. The products were then purified and enriched with 15 cycles of PCR. The final cDNA libraries were validated with a Fragment Analyzer (Advanced Analytical, Ankeny, IA, USA) and quantified with a KAPA qPCR kit (Kapa Biosystems, Wilmington, MA, USA).

For each sequencing lane of a flowcell V4, ten libraries were pooled in equal proportions, denatured with NaOH and diluted to 8,5 pM before clustering. Cluster formation, primer hybridisation and single end-read 50 cycle sequencing were performed on cBot and HiSeq2500 (Illumina, San Diego, CA, USA), respectively. Image analyses and base-calling were performed using the HiSeq Control Software (HCS) and Real-Time Analysis component (RTA). The quality of the data was assessed using FastQC from the Babraham Institute and the Illumina software SAV (Sequence Analysis Viewer) [69].

### RNA-seq data assembly and annotation

The read quality of the RNA-seq libraries was evaluated using FastQC [69]. Cleaned and filtered reads were de novo assembled using DRAP version 1.7 (de novo RNA-seq Assembly Pipeline) [70] with the Oases assembler [71] and filtered with at least one FPKM. The resulting contigs were aligned with NCBI blast (blastx program, version 2.2.26, e-value under 1e-5) on Refseq protein, Swissprot and Ensembl protein reference files from *Apis mellifera* and *Metaseiulus occidentalis* to retrieve the corresponding annotations. The contigs were also processed with rnammer (standard parameters, version 1.2) [72] to find ribosomal genes, with repeatmasker (−engine crossmatch -gccalc -species *Varroa destructor* parameters, version open-4-0-3) [73] to list the contained repeats and with interproscan (−-goterms –pathways parameters, version 4.8) [74] for gene ontology and structural annotation. Reads were realigned back to contigs with bwa (standard parameters, mem algorithm, version 0.7.12) [75]. The resulting sam files were compressed, sorted and indexed with samtools (view, sort and index programs, standard parameters, version 1.1) [76]. The contig expression counts were generated from the bam files with samtools (idxstats program, standard parameters, version 1.1) and merged with unix commands (cut, paste). The alignment files were then filtered for duplicates with samtools (rmdup program, standard parameters, version 1.1) before variant calling (SNPs and INDELs). The resulting bam files were processed with GATK (−glm BOTH parameter, version v3.0–0-g6bad1c6) following the best practices found on the GATK website [77]. All the results were uploaded in a RNAbrowse instance [78] and can be accessed from the web at http://ngspipelines.toulouse.inra.fr:9007/.

### Differential expression analysis

The table of raw mapped read counts per replicate (each of which is made up of 10 pooled mites) and per contig was built from the mapping statistics provided by samtools idxstats (Additional file 10). Differential analyses were performed using the DESeq2 Bioconductor package version 1.10.1 [79]. Briefly, DESeq2 performs the following steps: (1) calculate normalization factors for each sample to adjust for differences among library sizes; (2) estimate per-contig dispersions using a weighted local regression of log dispersions over log base means (fitType = “local”); (3) fit a negative binomial generalized linear model with a fixed effect for life-cycle stage for each contig; and (4) calculate per-contig Wald test statistics to identify significantly differentially expressed contigs. For each comparison tested in the differential analysis, *P*-values were corrected for multiple testing using the Benjamini-Hochberg control of the false discovery rate at 5% [80]. Heatmaps of the log normalized count *Z*-scores for differential contigs were produced using the pheatmap R package [81], and plots of the dynamic relative expression of contigs were produced using the tidyverse, in particular the ggplot2 R package [82].

The dataset was also screened for matching to known honeybee RNA viruses, including the following list: SBV - Sacbrood virus (NC_002066.1) [83], VDV-1 - *Varroa destructor* virus-1 also known as Deformed wing virus-B or DWV-B (NC_006494.1) [84], VDV-2 (KX578271.1) [85], VDV-3 (KX578272) [85], DWV-A (NC_004830.2) [86], BQCV - Black queen cell virus (NC_003784.1) [87], ABPV - Acute bee paralysis virus (NC_002548.1) [88], KBV - Kashmir bee virus (NC_004807.1) [89], CBPV - Chronic bee paralysis virus (NC_010711.1) [90], CBPV RNA-2 - Chronic bee paralysis virus RNA-2 (NC_010712.1) [90], LSV-1 - Lake sinai virus-1 (HQ871931.2) [91], LSV-2 - Lake sinai virus-2 (HQ888865.2) [91], ALPV - Aphid lethal paralysis virus (JX045858.1) [92], BSRV - Big sioux river virus ou *Rhopalosiphum padi* virus (NC_001874.1) [93], BeeMLV - Bee Macula-like virus (NC_027631.1) [94], VTLV - *Varroa* tymo-like virus (NC_027619.1) [94], IAPV - Israeli acute paralysis virus (NC_009025.1) [95], SBPV - Slow bee paralysis virus (NC_014137.1) [96], SBPV Harpenden - Slow bee paralysis virus Harpenden (GU938761.1) [96], BeeMLV PSU-1var - Bee macula-like virus isolate PSU-1var (KT162925.1) [94], LSV VBP022 - Lake Sinai virus strain VBP022 (KM886902.1) [97], LSV VBP166 - Lake sinai virus strain VBP166 (KM886903.1) [97], LSV VBP256 - Lake sinai virus strain VBP256 (KM886904.1) [97], LSV exp10 - Lake sinai virus strain exp10 (KM886905.1) [97]. The mean counts of all contigs for each species was averaged across the four pools of 10 mites as an estimate of virus abundance. Comparisons of estimated virus abundance between the different stages and conditions was then realised on the log mean contig counts by an ANOVA with least squares means (lsmeans R package version 2.26–3), with a Tukey adjustment (5%).

### Co-expression analysis

A co-expression analysis was performed for contigs identified as differentially expressed in at least one of the life-cycle stages (Young, Phor, Arrest, Pre-lay, Laying, Post-lay, Emerg) by calculating a likelihood ratio test of the full model (~stage) against the reduced model (~ 1), and retaining contigs with a BH-adjusted *P*-value < 0.10. Using the coseq Bioconductor package version 0.99.12 [98], a Gaussian mixture model was fit to the arcsine-transformed normalized profiles of differentially expressed contigs for *K* = 2, …, 50 clusters. Based on the Integrated Completed Likelihood (ICL) criterion for model selection [99], the model with *K* = 18 clusters was selected. However, because the model with *K* = 14 clusters was found to yield a similar clustering result (adjusted Rand index = 0.51) while being more parsimonious, it was instead retained for subsequent analyses. Contigs were assigned to clusters using the maximum a posteriori (MAP) rule.

### Enrichment analyses

The Gene Ontology (GO) annotations were retrieved from the swissprot and RefSeq protein annotations, and were formatted to fit the topGO custom format. GO enrichment analyses were performed for lists of differentially over- and under-expressed genes as well as for each co-expression cluster using a Fisher’s exact test as implemented in the topGO Bioconductor package version 2.22.0 [100]. The testing list was defined from the GO terms associated with differentially expressed contigs or each cluster, and the contig universe corresponded to the contigs expressed in adult mites.

## Additional files

Additional file 1: Distribution of contigs according to length. (XLSX 9014 kb)

Additional file 2: Contigs mapped per library. R204, R245, R41 and R78 correspond to the colony replicate and the last digit of the library ID to the life-cycle stage or condition: 1. Arrest, 2. Pre-lay, 3. Laying, 4. NR, 5. Emerg., 6.Young, 7. Male, 8. Phor, 9. Cage, 10. Post-lay. (XLSX 11673 kb)

Additional file 3: Graphical representation of contig annotation. (XLSX 106 kb)

Additional file 4: Contig description. (XLSX 19 kb)

Additional file 5: Heatmap of Pearson correlations between samples, based on log_10_ expression counts across contigs. The heatmap was produced using the pheatmap R package [81], and rows and columns were clustered using hierarchical clustering using the Euclidean distance and complete linkage. (PDF 193 kb)

Additional file 6: Lists of contigs differentially expressed in each pairwise comparison of mite life-cycle stages and conditions. Each worksheet reports results for a specific pairwise comparison. The columns include contig ID, log2 fold change, *P*-value and BH adjusted *P*-value, and contig description. (PDF 87 kb)

Additional file 7: Dynamic expression of contigs encoding vitellogenin and the large lipid transfer protein across the *Varroa* reproductive cycle. After averaging values for each contig across replicates in each stage, mean expression values are reported as log_2_ fold-change with respect to the Young stage. (PDF 89 kb)

Additional file 8: Dynamic expression of contigs encoding Halloween genes and ecdysone receptor across the *Varroa* reproductive cycle. After averaging values for each contig across replicates in each stage, mean expression values are reported as log_2_ fold-change with respect to the Young stage. (PDF 112 kb)

Additional file 9: Enriched GO terms for each pairwise comparison of mite life-cycle stages and conditions. Each worksheet reports results (biological process and molecular function) for a specific pairwise comparison. The columns indicate GO ID, GO term, total number of contigs in the GO category, number of contigs differentially expressed within the category, expected number of contigs and *P*-value. (XLSX 4646 kb)

Additional file 10: Raw counts of mapped reads per contig and sample. The contig expression corresponds to the number of reads aligned on the contig. Each column contains the expression measures of a contig for each of the different samples. In the sample names (library IDs), R204, R245, R41 and R78 correspond to the colony replicate and the last digit of the library ID to the life-cycle stage or condition: 1. Arrest, 2. Pre-lay, 3. Laying, 4. NR, 5. Emerg., 6.Young, 7. Male, 8. Phor, 9. Cage, 10. Post-lay. (PDF 14 kb)

Additional file 11: Co-expressed contigs during the adult mite cycle. The co-expression analysis was performed for contigs identified as differentially expressed in at least one of the life-cycle stages by performing a likelihood ratio test of the full model (~stage) versus the null model (~ 1). Cluster labels, as well as the normalized expression counts for each sample, are provided for all contigs included in the co-expression analysis. In the sample names (library IDs), R204, R245, R41 and R78 correspond to the colony replicate and the last digit of the library ID to the life-cycle stage or condition: 1. Arrest, 2. Pre-lay, 3. Laying, 5. Emerg., 6.Young, 8. Phor, 10. Post-lay. (XLSX 6591 kb)

Additional file 12: Enriched GO terms in clusters of contigs co-expressed during the mite cycle. Each worksheet reports results (biological process and molecular function) for a specific cluster. The columns indicate GO ID, GO term, total number of contigs in the GO category, number of contigs co-expressed within the category, expected number of contigs and *P*-value. (PDF 4 kb)

Additional file 13: List of contigs encoding genes from the RNAi pathways, sialome, and neurotransmitters and neuropeptides receptors identified from the co-expression analysis. Their expression patterns are shown in Fig. 7. (PDF 5 kb)

Additional file 14: Abundance of viruses across the reproductive cycle of foundress and different conditions of adult *Varroa*. Virus abundance is expressed as the mean number of counts for contigs that matched DWV (Deformed Wing virus), VDV-1 (*Varroa destructor* virus 1), VDV-2 (*Varroa destructor* virus 1) or Macula viruses in pools of 10 mites (*n* = 4 for each group). (XLSX 199 kb)

## Abbreviations

ABPV: Acute bee paralysis virus
ALPV: Aphid lethal paralysis virus
BeeMLV: Bee Macula-like virus
BQCV: Black queen cell virus
BSRV: Big sioux river virus
CBPV: Chronic bee paralysis virus
*CYP302A1*: Cytochrome P450-302a1/disembodied
*CYP307a1*: Cytochrome P450-307a1/spook
*CYP314A1*: Cytochrome P450-314a1/shade
CYP450: Cytochrome P450
DWV: Deformed wing virus
GO: Gene ontology
IAPV: Israeli acute paralysis virus
INDELs: Insertion or deletion of bases
KBV: Kashmir bee virus
L5: Day 5 of larval development
*lltp*: Large lipid transfer protein
LSV: Lake sinai virus
NaOH: Sodium hydroxide
P5: Day 5 of pupal development
P8: Day 8 of pupal development
P9: day 9 of pupal development
RNAi: RNA interference
RNA-seq: Ribonucleic acid sequencing
SBPV: Slow bee paralysis virus
SBV: Sacbrood virus
SNP: Single-nucleotide polymorphism
VDV: *Varroa destructor* virus
*vg*: Vitellogenin
VTLV: *Varroa* tymo-like virus
