# Supplementary figures and images for: Transcriptome profiling of the honeybee parasite Varroa destructor provides new biological insights into the mite adult life cycle
Source: BMC Genomics. 2018 May 4;19:328. doi: 10.1186/s12864-018-4668-z (PMC5936029; doi:10.1186/s12864-018-4668-z)

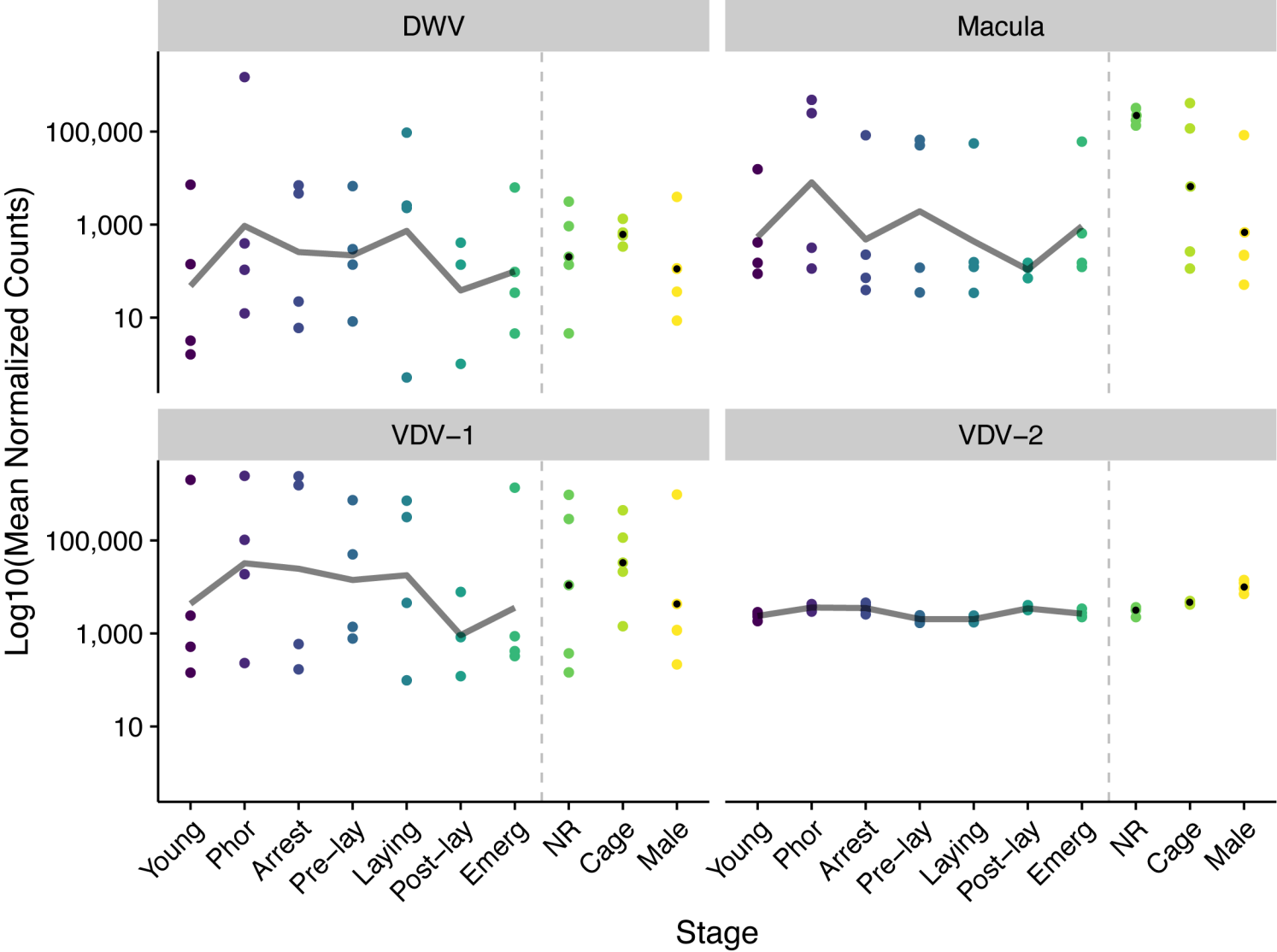

Supplement: Supplementary file 5 — Heatmap of Pearson correlations between samples, based on log10 expression counts across contigs. The heatmap was produced using the pheatmap R package [81], and rows and columns were clustered using hierarchical clustering using the Euclidean distance and complete linkage. (PDF 193 kb) [file 12864_2018_4668_MOESM14_ESM.pdf]

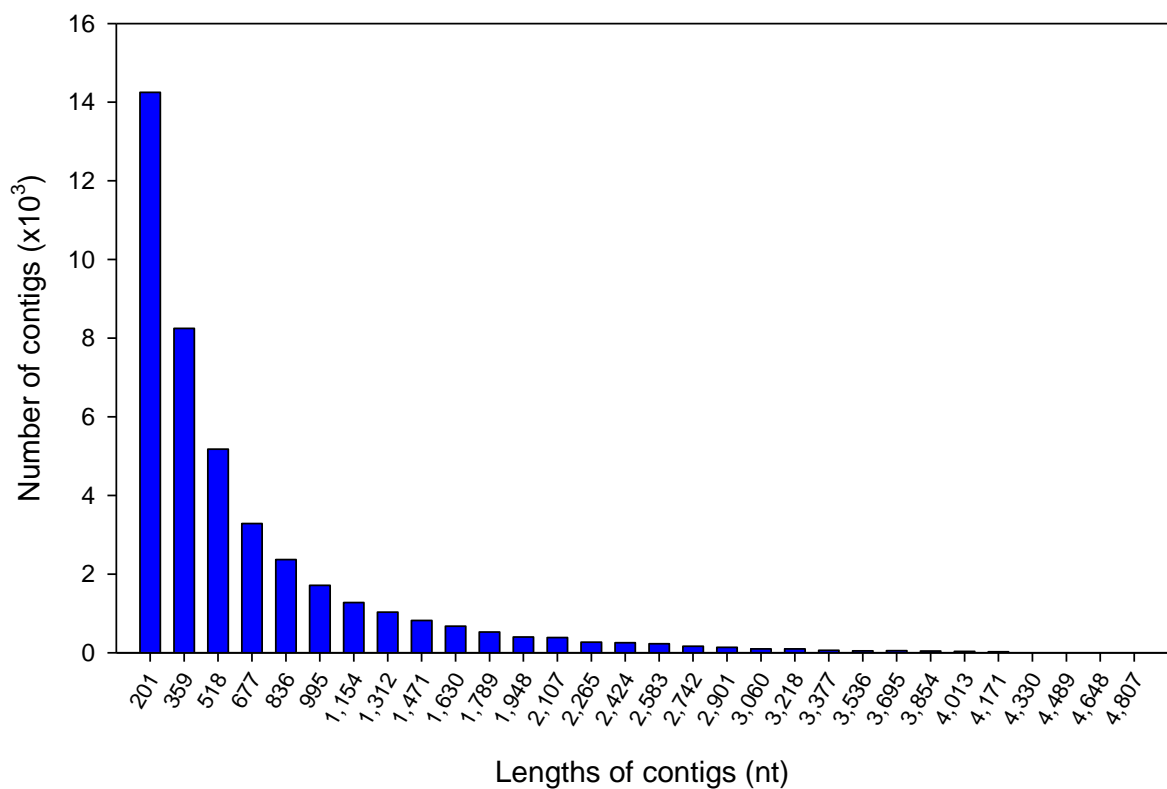

Supplement: Supplementary file 6 — Lists of contigs differentially expressed in each pairwise comparison of mite life-cycle stages and conditions. Each worksheet reports results for a specific pairwise comparison. The columns include contig ID, log2 fold change, P-value and BH adjusted P-value, and contig description. (PDF 87 kb) [file 12864_2018_4668_MOESM1_ESM.pdf]

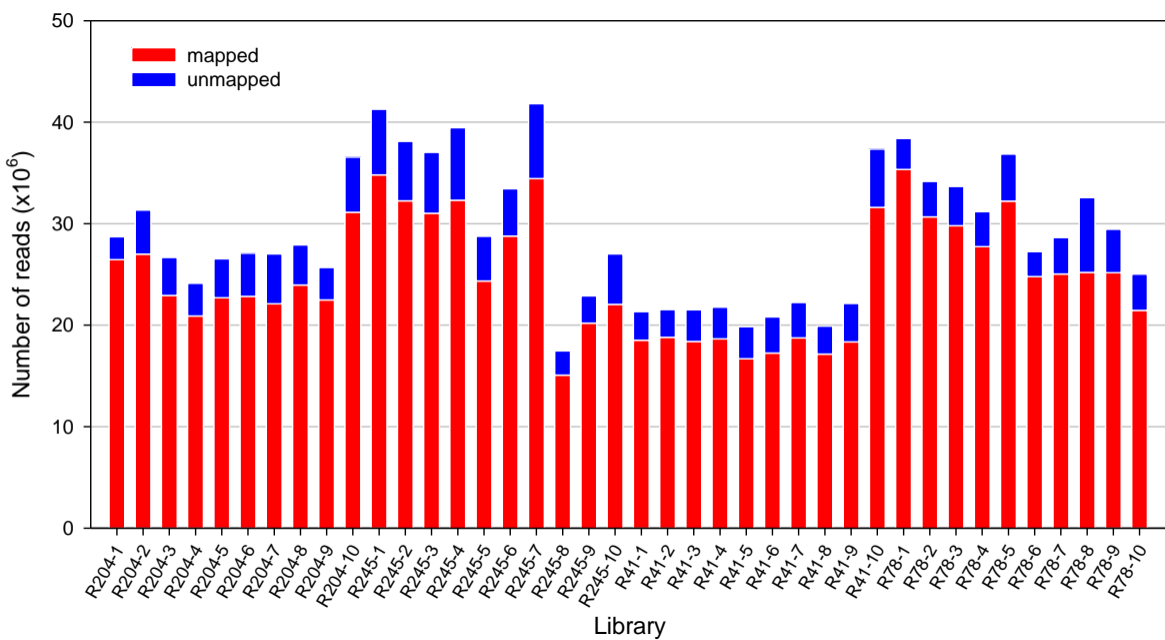

Supplement: Supplementary file 7 — Dynamic expression of contigs encoding vitellogenin and the large lipid transfer protein across the Varroa reproductive cycle. After averaging values for each contig across replicates in each stage, mean expression values are reported as log2 fold-change with respect to the Young stage. (PDF 89 kb) [file 12864_2018_4668_MOESM2_ESM.pdf]

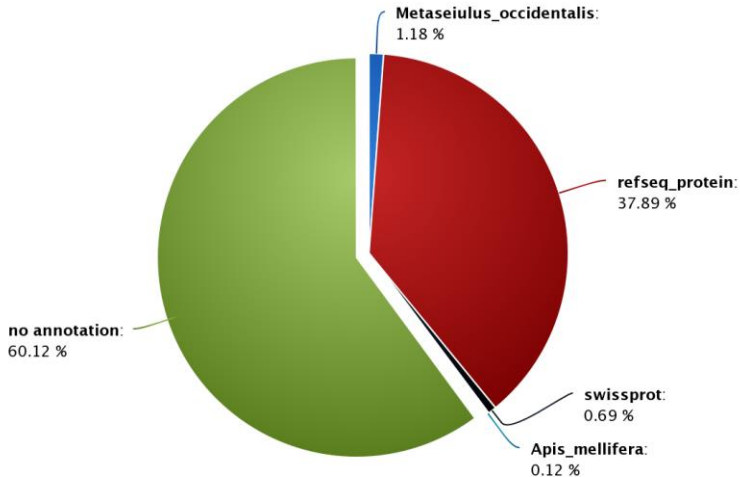

Supplement: Supplementary file 8 — Dynamic expression of contigs encoding Halloween genes and ecdysone receptor across the Varroa reproductive cycle. After averaging values for each contig across replicates in each stage, mean expression values are reported as log2 fold-change with respect to the Young stage. (PDF 112 kb) [file 12864_2018_4668_MOESM3_ESM.pdf]

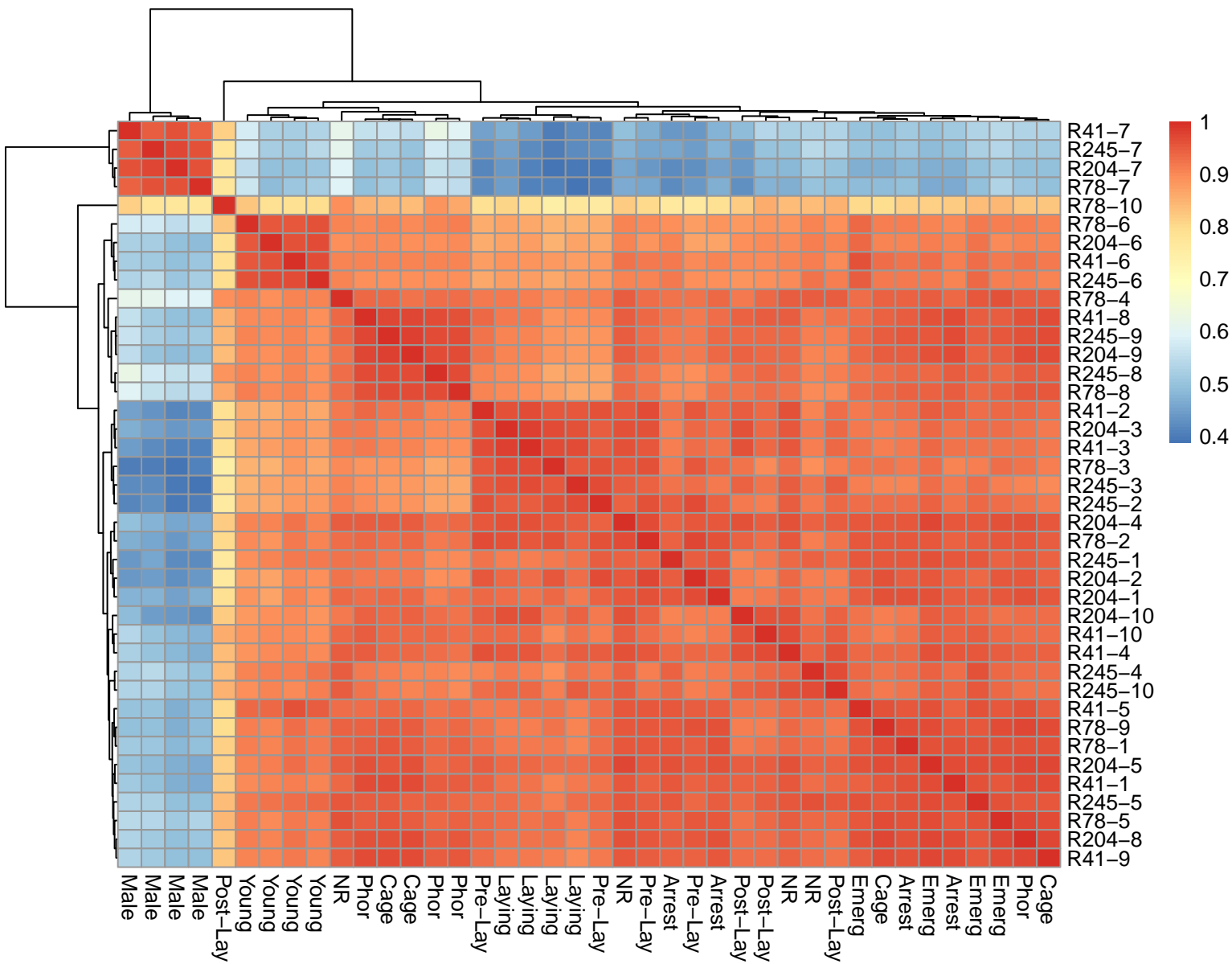

Supplement: Supplementary file 10 — Raw counts of mapped reads per contig and sample. The contig expression corresponds to the number of reads aligned on the contig. Each column contains the expression measures of a contig for each of the different samples. In the sample names (library IDs), R204, R245, R41 and R78 correspond to the colony replicate and the last digit of the library ID to the life-cycle stage or condition: 1. Arrest, 2. Pre-lay, 3. Laying, 4. NR, 5. Emerg., 6.Young, 7. Male, 8. Phor, 9. Cage, 10. Post-lay. (PDF 14 kb) [file 12864_2018_4668_MOESM5_ESM.pdf]

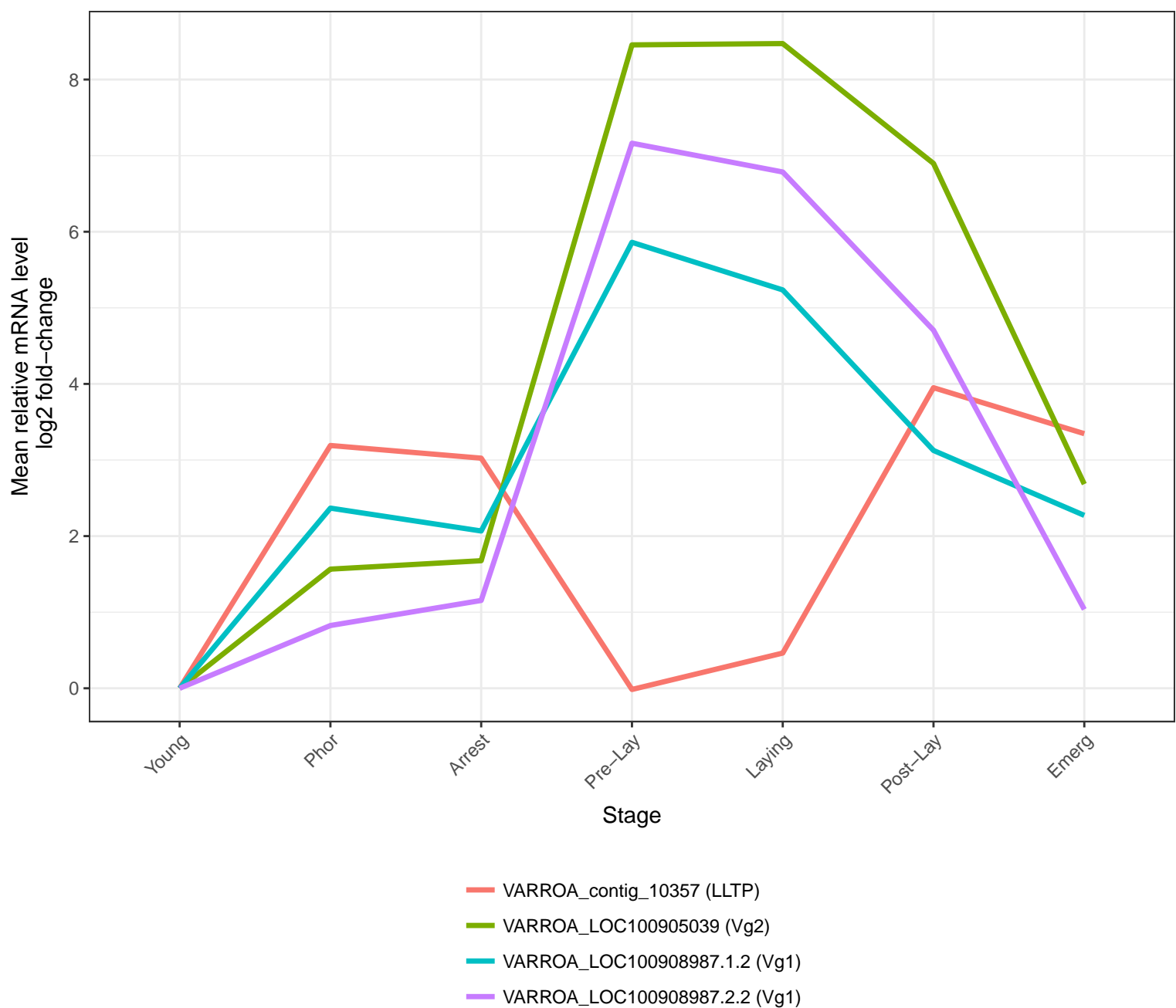

Supplement: Supplementary file 12 — Enriched GO terms in clusters of contigs co-expressed during the mite cycle. Each worksheet reports results (biological process and molecular function) for a specific cluster. The columns indicate GO ID, GO term, total number of contigs in the GO category, number of contigs co-expressed within the category, expected number of contigs and P-value. (PDF 4 kb) [file 12864_2018_4668_MOESM7_ESM.pdf]
