# Supplementary material for: Transcriptome profiling of the honeybee parasite Varroa destructor provides new biological insights into the mite adult life cycle
Source: BMC Genomics. 2018 May 4;19:328. doi: 10.1186/s12864-018-4668-z (PMC5936029; doi:10.1186/s12864-018-4668-z)
Supplement: Supplementary file 13 — List of contigs encoding genes from the RNAi pathways, sialome, and neurotransmitters and neuropeptides receptors identified from the co-expression analysis. Their expression patterns are shown in Fig. 7. (PDF 5 kb) [file 12864_2018_4668_MOESM8_ESM.pdf]

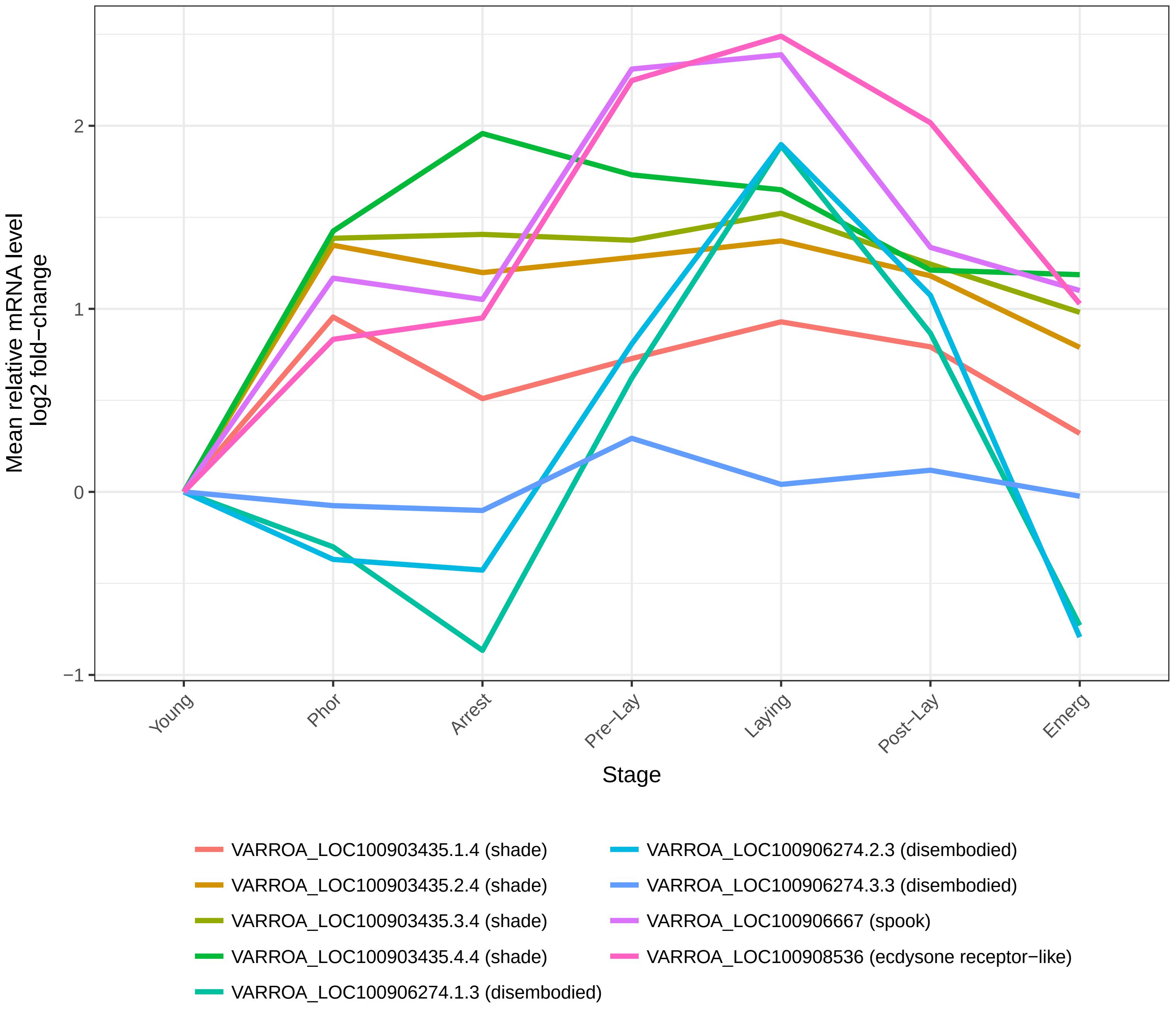
Mean relative mRNA level
log2 fold−change
2
1
0
−1
Young
Phor
Arrest
Pre−Lay
Laying
Post−Lay
Emerg
Stage
VARROA_LOC100903435.1.4 (shade)
VARROA_LOC100903435.2.4 (shade)
VARROA_LOC100903435.3.4 (shade)
VARROA_LOC100903435.4.4 (shade)
VARROA_LOC100906274.1.3 (disembodied)
VARROA_LOC100906274.2.3 (disembodied)
VARROA_LOC100906274.3.3 (disembodied)
VARROA_LOC100906667 (spook)
VARROA_LOC100908536 (ecdysone receptor−like)
